# The importance of childhood and adulthood aspects of gendered life for adult mental ill-health symptoms – a 27-year follow-up of the Northern Swedish Cohort

**DOI:** 10.1186/1471-2458-12-493

**Published:** 2012-07-02

**Authors:** Anna Månsdotter, Mikael Nordenmark, Anne Hammarström

**Affiliations:** 1 Department of Public Health Sciences, Karolinska Institutet, 171 76 STOCKHOLM, Sweden; 2 Department of Health Sciences, Mid Sweden University, 831 25 ÖSTERSUND, Sweden; 3 Department of Public Health and Clinical Medicine, Umeå University, 901 878, UMEÅ, Sweden

## Abstract

**Background:**

The increasing gender equality during the 20th century, mainly in the Nordic countries, represents a major social change. A well-established theory is that this may affect the mental health patterns of women and men. This study aimed at examining associations between childhood and adulthood gendered life on mental ill-health symptoms.

**Methods:**

A follow-up study of a cohort of all school leavers in a medium-sized industrial town in northern Sweden was performed from age 16 to age 42. Of those still alive of the original cohort, 94% (n = 1007) participated during the whole period. Gendered life was divided into three stages according to whether they were traditional or non-traditional (the latter includes equal): childhood (mother’s paid work position), adulthood at age 30 (ideology and childcare), and adulthood at age 42 (partnership and childcare). Mental ill-health was measured by self-reported anxious symptoms (“frequent nervousness”) and depressive symptoms (“frequent sadness”) at age 42. The statistical method was logistic regression analysis, finally adjusted for earlier mental ill-health symptoms and social confounding factors.

**Results:**

Generally, parents’ gendered life was not decisive for a person’s own gendered life, and adulthood gender position ruled out the impact of childhood gender experience on self-reported mental ill-health. For women, non-traditional gender ideology at age 30 was associated with decreased risk of anxious symptoms (76% for traditional childhood, 78% for non-traditional childhood). For men, non-traditional childcare at age 42 was associated with decreased risk of depressive symptoms (84% for traditional childhood, 78% for non-traditional childhood). A contradictory indication was that non-traditional women in childcare at age 30 had a threefold increased risk of anxious symptoms at age 42, but only when having experienced a traditional childhood.

**Conclusion:**

Adulthood gender equality is generally good for self-reported mental health regardless of whether one opposes or continues one’s gendered history. However, the childcare findings indicate a differentiated picture; men seem to benefit in depressive symptoms from embracing this traditionally female duty, while women suffer anxious symptoms from departing from it, if their mother did not.

## Background

The steps towards improved gender equality during the 20th century, predominantly in the Nordic countries, represent a major social change. A well-accepted theory by now is that this has affected, and will affect, the health patterns of women and men [1].

Generally, women suffer more than men in qualitative aspects of ill-health, such as self-reported physical and psychological suffering, while men suffer more than women in quantitative aspects of ill-health, such as lifestyles leading to earlier deaths. When both aspects are considered, some measures (DALYs, QALYs, etc.) suggest that men are worst off [2], while others (for example overall self-rated health) suggest that men and women are quite equally harmed [3]. The general picture regarding mental health problems, such as feeling nervous and anxious, sad and depressed, is that men are better off than women [4-6].

There are reasons to assume that a gender-relational approach [7] is helpful when analysing differences in mental health between men and women [8,9]. The focus in this approach is on gender relations in all important spheres of life. Gender relations in family life include women being supposed to care for the children and household, which contributes to their societal subordination in terms of power and incomes [10]. The combination of caring duties and lack of resources may lead to worries and despair, and hence represent one explanation for the excess of mental health problems in women compared to men [11]. Correspondingly, gender relations in the sphere of working life are related to the view of men as breadwinners, and of men having higher incomes, which contributes to their societal dominance. A high degree of power, resources, and influence over life is generally a mental health promoting factor, which may explain why men have better mental health than women [12].

Women who are gender-equal in family and working life are therefore expected to have better mental health than traditional women, while gender-equal men could be expected to have worse mental health than traditional men [13,14]. However, the picture tends to be more complex.

Even though money and prestige generally predict good lifetime health, the unpaid and unrewarded caring duties also hold health-promoting aspects such as intimacy, emotions, and cautiousness [15]. It could also be the case that expanding one’s life, for example, by adding paid work for women and caring duties for men, is beneficial for the mental health of both genders [16-19]. In other words, having more than one identity (or social role) to lean upon may prepare individuals for tackling difficulties at work, in partnership, with the children, etc. The benefits of gender equality for mental health among both women and men are also supported by research showing that individuals’ relative position in terms of resources may be more decisive for lifetime health than their absolute level of resources [20]. However, since women tend to enter the public sphere of earning before men enter the domestic sphere of childcare, one must consider the risk of mental health problems mainly among women due to exhausting multiple demands [21-27].

Further illustration of the complex relationship between gender equality and mental health is related to the social and cultural constructions of femininities and masculinities [8,28]. Hence, trading off gendered traditions with power and independence (for women), and with caring and dependence (for men), involves risks of vulnerability and guilt [29]. This also helps to explain why supporting a gender-equal society in general may differ from actually practising gender-equal relations at work, in the family, and during spare time. In other words, embracing a gender-equal ideology but continuing a gender-unequal practice may be less detrimental for mental health than a solid gender-equal life [30]. Yet, the number of reasonably gender-equal partnerships and contexts is increasing [31,32]. Hence, individuals who reject or fail to achieve gender equality in daily life may feel unusual or frustrated, which may also entail problems [33,34].

The theories of benefits from expanding one’s life with additional roles [16], of damaging stress from adding too many roles [35], and of gender ideology versus practice [30] could be referred to role theory. Generally, this explains human activity in terms of expectations held by the individual and/or other people [36]. Within role theory, a number of concepts have emerged: role confusion, which refers to dilemma in deciding which role to take on; role embracement, which refers to holding a role so much that the self disappears; and role strain, which refers to tensions from incompatible expectations within a role. When examining mental ill-health, the latter can be further divided into, for example, role ambiguity (incomplete guidance for a role), role incongruence (required change in transition of a role), and role over-qualification (unused personal resources in a role) [19,37]. In the context of gender equality, however, it must be considered that feminist researchers have criticized role theory for assuming complementarity and harmony between men and women, which masks unequal social conditions [38]. The criticism includes that internal conflicts are focused in a way that suggests that the lack of gender equality is the fault of women, rather than the source being an imbalance of power between the genders.

Finally, an individual’s history of gendered life (for example, how the parents divided duties during childhood) may play a role for the consequences of gender equality in adult age for lifetime health. In a recent study Kroska and Elman [39] found general support for the hypothesis that individuals seek to adjust in order to maintain gender ideology and practice over their lifetime. However, the mother’s employment during a person’s childhood was positively related to egalitarian (gender-equal) views and practices only for women, and the mental health consequences of maintaining, or not maintaining, gendered history were not studied. Nevertheless, it seems reasonable to assume that childhood experiences of gendered life are likely to affect gendered patterns in adulthood, and that continuing or opposing one’s gendered tradition in adult life may have a unique impact on mental health prospects [40,41].

### Aims and assumptions

This study aims at examining the importance of gendered life in childhood and adulthood for self-reported mental ill-health at age 42 in the Northern Swedish Cohort. Ultimately, it reports on whether the combination of childhood gender experience and adulthood gender position may have an impact on anxious and depressive symptoms. Our expectations are that: the net effect of a less gender-traditional life on mental health is positive for both genders, though women are likely to benefit more than men; continuing one’s parents’ history is more beneficial for mental health than opposing it, though adulthood position has a stronger impact than childhood experience.

## Methods

### Participants

This cohort study includes all school leavers from the last year of compulsory school (age 16) in all schools in a medium-sized industrial town in the north of Sweden in 1981, known as the Northern Swedish Cohort. The attrition rate was extremely low. At the 26-year follow-up 93.9 % (n =1007) of those still alive of the original cohort (n = 1083) continued to participate. For this study the sample of the cohort was based on the 26-year follow-up including 481 women and 526 men.

### Procedure

All participants were investigated at ages 16, 18, 21, 30 and most recently at age 42 (in autumn 2007) with a comprehensive questionnaire. More detailed information about the cohort has been published elsewhere [42]. The questionnaire consisted of about 90 questions regarding family and working life as well as health status. The same questionnaire was repeated at the follow-ups. The questionnaire was derived from well-used questionnaires such as the Swedish national Survey of Living Conditions [43], and the Low-Income Study [44].

The study has been approved by the Regional Ethics Vetting Board in Umeå.

### Measuring gendered life

The indicators of gendered life are conceptually divided into either a traditional or a non-traditional position [15]. The concept of *traditional* refers to supporting the idea, or practising the situation, where women dominate the private and men the public sphere of life [10]. The concept of *non-traditional* refers to challenging this, either by supporting or practising gender equality or by shifting the gendered tradition (e.g. the father dominates versus the mother in childcare).

*Childhood gendered life* (i.e. gendered life in the family of origin) was indicated by the mother’s participation in the labour market during childhood. The self-reported information was obtained from the survey of 1981 (at age 16) and categorised into traditional: “mother no paid work”, and non-traditional: “mother part/full-time paid work”; the inclusion of *part-time* in the non-traditional category was due to the gendered practice regarding women’s labour market participation in Sweden in 1965–1981 [45], and intended to secure statistical power in the combined analyses of childhood and adulthood gender.

*Adulthood gendered life* was analysed with two indicators (gendered ideology and childcare practice) from the survey of 1995 (at age 30) and two indicators (gendered partnership and childcare practice) from the survey of 2007 (at age 42):

 · Gendered ideology at age 30 was measured with a scale indicating support for societal gender equality ranging from 1 (fully supporting a gender-equal society) to 10 (fully rejecting a gender-equal society), and categorised into traditional: “ranking 4–10”, and non-traditional “ranking 1–3”; the inclusion of *just three rankings* in the non-traditional category was due to our interpretation of gendered ideology in Sweden around the year 1995 [39], and the patterns found in data.

 · Gendered partnership at age 42 was based on a five-scale question regarding the level of overall gender equality in one’s relationship with a partner, and categorised into traditional: “tolerably/hardly/not equal”, and non-traditional: “entirely/fairly equal”; the exclusion of *tolerably equal* from the non-traditional category was due to the assumption that this refers to traditional inequality, which in terms of partnership fits the idea of “love” [7], and the patterns found in data.

 · Gendered childcare at age 30 and 42 was based on a five-scale question regarding the division of childcare responsibility, and categorised into traditional: “all/most” (women) and “less/nothing” (men), and non-traditional: “similar/less/nothing” (women) and “similar/most/all” (men); this dichotomisation is clear-cut in principle, although highly gendered in terms of how women and men interpret the various categories.

The general reason for studying gendered life at three points of time is that mental health consequences of gender (in)equality may arise in the short as well as the long run [46]. The specific reason for considering the division of childcare at age 30 (mean age first child: 5 years women; 4 years men) and at age 42 (mean age first child: 17 years women; 16 years men) was that parents’ division of childcare duties is likely to vary with the age of the child, and have different relationships with mental ill-health [14].

Ultimately, the one indicator of childhood gendered life was combined with each of the four indicators of adulthood gendered life into four combinations of gender positions (traditional childhood/traditional adulthood, traditional childhood/non-traditional adulthood, non-traditional childhood/traditional adulthood, non-traditional childhood/non-traditional adulthood) and four combinations of gender indicators (mother’s work/own ideology, mother’s work/own earlier childcare, mother’s work/own partnership, mother’s work/own later childcare).

### Measuring self-reported mental ill-health

The outcomes consisted of two variables indicating mental ill-health from the survey of 2007; anxious symptoms: “often/always nervous the last 12 months” (in contrast to “never/now and then”), and depressive symptoms: “often/always sad the last 12 months” (in contrast to “never/now and then”). Both questions were derived from the validated Swedish Survey of Living Conditions (Statistics Sweden).

### Baseline/confounders

In order to control for base-line mental ill-health (and to address the changes in mental ill-health from age 16 until age 42) we included self-reported anxious and depressive symptoms at age 16. The variables were measured and dichotomised in exactly the same way as the outcome variables. The following confounders were included: “perceived childhood class” at age 16 measured as working, middle, or upper class (due to associations with gendered life and mental health), “divorced or dead parents” at age 16 (due to associations with mother’s paid work position and own mental health), “educational level at age 30 measured as mandatory, upper secondary or higher schooling” (due to associations with gendered life and influence on mental health), “being married/cohabiting” at age 30 (since this may be associated with gendered childcare), and “number of children” at age 30 (since this may be associated with gendered partnership).

### Statistical analyses

The first step was to examine the patterns of gendered life, mental ill-health symptoms at baseline, confounders and mental ill-health symptoms at age 42, in total and by gender (the latter included reports on p-values from chi-square tests). The second step was to examine the relationship between the five indicators of gendered life and the two measures of mental ill-health symptoms in bivariate logistic regression analyses. The third step was to examine the distribution of the combinations of the one childhood (traditional/non-traditional) and four adulthood (traditional/non-traditional) indicators of gendered life by gender. This included reports on p-values from chi-square tests for the distribution of women (among all women) versus men (among all men), regarding the four possible childhood/adulthood positions (traditional/traditional, traditional/non-traditional, non-traditional/traditional, non-traditional/non-traditional), by a cross tabulation for each indicator of gendered life (ideology, partnership, childcare at age 30, childcare at age 42).

The fourth step was to examine the relationship between combinations of childhood/adulthood gendered life and self-reported mental ill-health outcomes by multivariate logistic regression modelling. The analyses were performed to report the relative risk among those categorised as traditional childhood but non-traditional adulthood, non-traditional childhood but traditional adulthood, and non-traditional childhood as well as adulthood, versus those categorised as traditional childhood as well as adulthood. The results presented were crude and adjusted for baseline anxious and depressive symptoms, childhood class, divorced/dead parents, educational level, married/cohabiting (in the analyses of adult childcare), and number of children (in the analyses of adult partnership), in total and by gender.

The logistic regression analyses were reported in odds ratios (OR), which regarding the outcomes of depressive and anxious symptoms were assumed to estimate the relative risk (RR). The second and fourth steps were complemented with a test of interaction between gender and gendered life on self-reported mental ill-health symptoms. All analyses were performed by SPSS version 18, and statistical significance was reported as p-values or confidence intervals (CI) at the 5% level.

## Results

Half of the respondents had a gender-traditional childhood in terms of mothers not working outside the home during childhood (Table 1). The adulthood indicators report that 27.3% had a gender-traditional ideology, 60.6% a gender traditional childcare division at age 30, 19.7% a traditional partnership, and 41.9% a traditional childcare division at age 42. On average, 7.1% of the respondents report anxious symptoms whilst 9.9% report depressive symptoms during the last year. Both anxious and depressive symptoms were more common among respondents without a partner at age 42 (12.9% and 17.4%, respectively) than among those with a partner (5.5% and 7.5%, respectively), and more common among respondents without children at age 42 (8.0% and 10.8%, respectively) than among those with children (6.9% and 9.4%, respectively) (figures not shown in table). The statistically significant differences between the genders concern: traditional gender ideology (men dominate), traditional childcare (women dominate both time points), traditional partnership (women dominate), experiencing anxious and depressive symptoms last year (women dominate both time points), perceived working class (men dominate), and not being married/cohabiting and not having children (men dominate). The most remarkable gender gap concerns childcare at age 42: 56.7% of women report a traditional division in terms of doing most/all, while only 25.7% of men report doing less/nothing.

**Table 1:** Distribution of the dependent and independent variables (%) in total and by gender; p-values for chi-square tests regarding the distribution between women and men

	**Number of respondents**	**Total**	**Women**	**Men**	***p-value chi-square women/men distribution***
Gendered life:					
Traditional childhood: (mother no paid work)	993	49.8	50.3	49.4	0.778
Traditional ideology (age 30): (indifferent/against gender equality)	990	27.3	19.2	34.6	<0.001
Traditional childcare (age 30): (Women: most/all Men: less/nothing)	639	60.6	67.0	52.8	<0.001
Traditional partnership (age 42): (tolerably/hardly/not equal)	761	19.7	23.1	16.3	0.019
Traditional childcare (age 42): (Women: most/all Men: less/nothing)	790	41.9	56.7	25.7	<0.001
Health baseline:					
Anxious symptoms last year (age 16): (often/always)	1001	1.4	2.3	0.6	0.020
Depressive symptoms last year (age 16): (often/always)	1004	6.4	10.6	2.5	<0.001
Social confounding:					
Perceived working class (age 16)	974	15.9	12.9	18.7	0.013
Divorced/dead parents (age 16)	1007	22.8	22.5	23.2	0.780
Mandatory schooling (age 30)	963	8.7	7.7	9.7	0.279
Not married/cohabiting (age 30)	971	23.0	19.7	26.1	0.019
No children (age 30)	969	17.9	11.7	23.7	<0.001
Health outcome:					
Anxious symptoms last year (age 42): (often/always)	968	7.1	8.9	5.4	0.035
Depressive symptoms last year (age 42): (often/always)	968	9.9	14.0	6.0	<0.001

The bivariate analyses (Table 2), for both genders, demonstrate no statistically significant associations between reporting a gender non-traditional childhood and aspects of adulthood gendered life. Further, neither a non-traditional childhood nor non-traditional ideology and childcare at age 30 were associated with self-reported mental ill-health. However, a non-traditional partnership at age 42 was associated with decreased risk of anxious symptoms (0.45) and non-traditional childcare at age 42 was associated with decreased risks of anxious (0.52) as well as depressive (0.53) symptoms, compared to the traditional counterparts. When stratified by gender, women, but not men, benefit from a more equal partnership (0.42) and men, but not women, benefit from more equal childcare (0.32) in terms of anxious symptoms. Yet, no statistically significant interaction was demonstrated between gender and gendered life indicators on self-reported mental ill-health. Further stratification shows that non-traditional childcare among men at age 42 is associated with a massive risk decrease of anxious symptoms (0.04) among those *not* having a partner (figures not shown in table).

**Table 2:** Bivariate analyses: associations between indicators of childhood/adulthood gendered life, and childhood gendered life and health outcomes; in total and by gender, odds ratios (OR), 95 % confidence intervals (CI) in brackets

**Childhood gender & Health outcome:Childhood gender & Adulthood gender:**	**OR Traditional childhood**	**OR Anxious symptoms(age 42)**	**OR Depressive symptoms(age 42)**
Traditional (reference) Non-traditional childhood	1 –	1 1.09 (0.66–1.80) Women: 1.35 (0.71–2.57) Men: 0.82 (0.37–1.80)	1 1.09 (0.71–1.66) Women: 1.37 (0.81–2.34) Men: 0.72 (0.34–1.52)
Traditional (reference) Non-traditional ideology (age 30)	1 1.22 (0.92–1.62) Women: 1.42 (0.88–2.29) Men: 1.10 (0.76–1.58)	1 0.62 (0.37–1.03) Women: 0.37 (0.19–0.74) Men: 0.90 (0.40–1.99)	1 1.04 (0.64–1.70) Women: 0.80 (0.42–1.52) Men: 1.00 (0.45–2.20)
Traditional (reference) Non-traditional childcare (age 30)	1 0.81 (0.59–1.12) Women: 0.75 (0.48–1.18) Men: 0.91 (0.57–1.45)	1 1.35 (0.73–2.48) Women: 1.50 (0.71–3.18) Men: 1.48 (0.50–4.39)	1 0.76 (0.45–1.30) Women: 0.93 (0.49–1.76) Men: 0.75 (0.28–2.03)
Traditional (reference) Non-traditional partnership (age 42)	1 0.95 (0.67–1.37) Women: 0.99 (0.61–1.60) Men: 0.94 (0.55–1.63)	1 0.45 (0.24–0.87) Women: 0.42 (0.19–0.90) Men: 0.64 (0.17–2.41)	1 0.70 (0.38–1.30) Women: 0.63 (0.32–1.24) Men: 2.61 (0.34–20.13)
Traditional (reference) Non-traditional childcare (age 42)	1 1.04 (0.78–1.39) Women: 0.98 (0.66–1.45) Men: 1.38 (0.86–2.20)	1 0.52 (0.30–0.92) Women: 0.88 (0.44–1.74) Men: 0.32 (0.12–0.88)	1 0.53 (0.33–0.85) Women: 0.83 (0.47–1.46) Men: 0.47 (0.18–1.28)

No consistent picture appears when childhood and adulthood gendered life are combined (Table 3). The most frequent combinations were: traditional childhood/non-traditional ideology at age 30 (37.8%), traditional childhood/traditional childcare at age 30 (31.4%), non-traditional childhood/non-traditional partnership at age 42 (41.0%), and traditional and non-traditional childhood/non-traditional childcare at age 42 (both 29.3%). Further, the differences in distributions for women and men are statistically significant for all combinations, except for childhood gendered life and adulthood gendered partnership. An illustration of this gender gap is that 29.2% of the women report the combination of having a traditional childhood and a traditional childcare division at age 30 (i.e. doing most/all), while the corresponding figure for men is 10.5% (i.e. doing less/nothing).

**Table 3:** Distributions (%) by combination of childhood (traditional/non-traditional) and adulthood (traditional/non-traditional); p-values for chi-square tests regarding the distribution of women and men, in the four combinations of childhood/adulthood position, for the four indicators of gendered life

**Adulthood gender: Childhoodgender:**	**Ideology age 30: Traditional**	**Ideology age 30: Non-traditional**	**Childcare age 30: Traditional**	**Childcare age 30: Non-traditional**	**Partnership age 42: Traditional**	**Partnership age 42: Non-traditional**	**Childcare age 42: Traditional**	**Childcare age 42: Non-traditional**
Traditional	Women: 8.0 Men: 16.5	Women: 43.2 Men: 33.0	Women: 36.1 Men: 25.7	Women: 15.5 Men: 22.2	Women: 11.9 Men: 8.0	Women: 39.1 Men: 39.2	Women: 29.2 Men: 10.5	Women: 22.8 Men: 36.5
Non-traditional	Women: 10.2 Men: 17.9	Women: 38.6 Men: 32.6	Women: 30.8 Men: 26.8	Women: 17.6 Men: 25.4	Women: 11.3 Men: 8.5	Women: 37.7 Men: 44.3	Women: 26.7 Men: 15.0	Women: 21.3 Men: 38.1
Subpopulation		n = 970		n = 625		n = 746		n = 777
p-value chi-square women/men distribution:		< 0.001		0.003		0.102		< 0.001

The general observation from the fully adjusted combinational analyses is that there are few statistically significant results (Table 4). For women, however, reporting a non-traditional ideology (supporting societal gender equality) involves decreased risks of anxious symptoms at 76% if combined with a traditional childhood (mother no paid work), and at 78% if combined with a non-traditional childhood (mother part/full-time paid work), compared to those traditional in childhood as well adulthood. For men, reporting a non-traditional childcare division at age 42 involves decreased risks of depressive symptoms at 84% if combined with a traditional childhood, and at 78% if combined with a non-traditional childhood. The only statistically significant interaction on this subject of gendered life and gender on mental ill-health regard childhood and childcare at age 42 on depressive symptoms.

**Table 4:** **Associations between four combinations of childhood (one indicator) and adulthood (four indicators) gendered life for women and men, numbers (n), crude and adjusted**^**Ω**^**odds ratios (OR), 95 % confidence intervals (CI) in brackets**

	**OR**				**OR**			
	**Anxious symptoms (often/always)**				**Depressive symptoms(often/always)**			
	**Women**	**Women**	**Men**	**Men**	**Women**	**Women**	**Men**	**Men**
	**Crude**	**Ω**	**Crude**	**Ω**	**Crude**	**Ω**	**crude**	**Ω**
Childhood gender /	n=449	n=431	n=481	n=454	n=448	n=431	n=482	n=455
Adult ideology (age 30):								
Traditional/Traditional (ref)	1	1	1	1	1	1	1	1
Traditional/Non-traditional	0.34	0.24	0.64	0.81	0.87	0.67	0.68	1.09
	(0.120.98)	(0.08–0.75)	(0.21–1.90)	(0.22–3.00)	(0.31–2.44)	(0.22–2.00)	(0.25–1.86)	(0.31–3.82)
Non-traditional/Traditional	1.26	0.67	0.58	0.61	1.56	1.26	0.36	0.49
	(0.40–3.92)	(0.19–2.37)	(0.16–2.13)	(0.13–2.81)	(0.47–5.12)	(0.35–4.50)	(0.09–1.45)	(0.10–2.49)
Non-traditional/Non-traditional	0.42	0.22	0.64	0.98	0.99	0.81	0.61	1.06
	(0.15–1.20)	(0.07–0.70)	(0.22–1.93)	(0.26–3.62)	(0.35–2.80)	(0.27–2.41)	(0.22–1.71)	(0.29–3.88)
Childhood gender/	n = 333	n = 316	n = 278	n = 266	n = 332	n = 316	n = 278	n = 266
Adult childcare (age 30)								
Traditional/Traditional (ref)	1	1	1	1	1	1	1	1
Traditional/Non-traditional	2.98	3.09	1.45	2.25	1.09	1.10	0.79	0.98
Non-traditional/Traditional	(0.95–9.36)	(0.90–10.62)	(0.37–5.65)	(0.42–12.00)	(0.42–2.85)	(0.38–3.23)	(0.24–2.63)	(0.24–4.12)
Non-traditional/Non-traditional	2.34	1.78	0.46	0.71	1.42	1.50	0.38	0.31
	(0.83–6.58)	(0.58–5.48)	(0.08–2.58)	(0.10–4.85)	(0.67–3.00)	(0.65–3.46)	(0.09–1.53)	(0.06–1.74)
Non-traditional/Non-traditional	2.17	0.94	0.72	1.23	1.26	1.03	0.26	0.32
	(0.67–7.04)	(0.22–4.01)	(0.15–3.33)	(0.22–6.93)	(0.52–3.08)	(0.36–2.69)	(0.05–1.28)	(0.06–1.76)
Childhood gender/	n = 371	n = 355	n = 373	n = 355	n = 370	n = 355	n = 374	n = 355
Adult partnership (age 42)								
Traditional/Traditional (ref)	1	1	1	1	1	1		
Traditional/Non-traditional	0.46	0.50	1.23	0.97	0.52	0.53		
	(0.14–1.47)	(0.15–1.75)	(0.14–10.64)	(0.08–11.27)	(0.20–1.41)	(0.181.53)		
Non-traditional/Traditional	1.30	1.14	1.93	1.54	0.88	0.72		
	(0.36–4.63)	(0.27–4.86)	(0.17–22.50)	(0.09–25.32)	(0.27–2.88)	(0.192.69)		
Non-traditional/Non-traditional	0.60	0.48	0.72	0.93	0.73	0.78		
	(0.19–1.86)	(0.14–1.68)	(0.08–6.72)	(0.08–11.01)	(0.28–1.90)	(0.282.20)		
Childhood gender/	n = 403	n = 386	n = 370	n = 354	n = 402	n = 386	n = 371	n=355
Adult childcare (age 42)								
Traditional/Traditional (ref)	1	1	1	1	1	1	1	1
	0.75	0.82	0.21	0.24	1.09	1.49	0.17	0.16
Traditional/Non-traditional	(0.26–2.14)	(0.27–2.44)	(0.05–0.81)	(0.04–1.25)	(0.48–2.49)	(0.61–3.66)	(0.04–0.62)	(0.03–0.82)
	1.34	1.15	0.40	0.44	1.66	1.90	0.10	0.10
Non-traditional/Traditional	(0.55–3.24)	(0.45–2.98)	(0.09–1.78)	(0.08–2.45)	(0.79–3.47)	(0.84–4.32)	(0.01–0.88)	(0.01–1.01)
	1.25	0.88	0.20	0.25	1.07	1.20	0.24	0.22
Non-traditional/Non-traditional	(0.48–3.22)	(0.30–2.60)	(0.05–0.78)	(0.05–1.17)	(0.46–2.48)	(0.46–3.15)	(0.07–0.81)	(0.05–0.91)

Ω adjusted for baseline anxious and depressive symptoms (age 16), perceived childhood class (age 16), divorced/dead parents (age 16), three levels of education (age 30), being married/cohabiting (age 30, in the childcare analyses), and number of children (age 30, in the partnership analysis).

## Discussion

We have performed a study on associations between childhood gendered life (mother’s paid work position during childhood), adulthood gendered life (ideology and childcare at the age of 30; partnership and childcare at age 42), and self-reported mental ill-health (anxious and depressive symptoms at age 42) based on the Northern Swedish Cohort. Gendered life was consequently divided into either traditional or non-traditional, the latter including equality. Ultimately, the study aimed at examining whether the combination of a gender (non-) traditional childhood and a gender (non-)traditional adulthood matters for mental ill-health symptoms for women and for men.

### Descriptive findings

The study confirms the generally reported gender gap in self-reported aspects of mental health; the women versus men excess at baseline (age 16) and follow-up (age 42) [4-6].

It also confirms well-established understandings of self-reported measures of gendered life; men dominate in reporting a gender-traditional ideology, whilst women dominate in reporting gender-traditional childcare as well as partnership. These findings comprise the different “standards” that women and men have regarding gender equality, which has been reported in earlier studies [30,34]. The large variances on average, from 19.7% reporting a traditional partnership to 60.6% reporting traditional early childcare, also confirm that steps towards gender equality vary with the gendered aspect measured [14].

The bivariate analyses did not show that the mother’s paid work position during childhood is associated with a person’s own gender position in adulthood, which supports earlier research on men [39]. In fact, there is a tendency that individuals who support societal gender equality are more likely (1.22, CI: 0.92–1.62) to report a gender-traditional childhood. Naturally, this may include socioeconomic confounding; during the 1970s, for example, maternal education was likely to increase incentives for paid work and the child’s educational ambition, which generally increases support for a gender-equal society. A more provocative interpretation is that childhood experiences of “missing mum at home” are transformed into an adult claim that women are connected to the private sphere of life [10].

It was also shown that childhood gendered life was not associated with adult mental ill-health symptoms. One explanation could be that the former was solely indicated by the mother’s paid work position, and not the relative caring and breadwinning positions of the parents. Even though the 1970s in Sweden was a decade of gender equality reforms [47], it could also be the case that individuals with traditional as well as non-traditional childhood felt confidently “normal” and hence not mentally affected by experiencing the one or another [33].

Further, reporting non-traditional partnership and childcare at age 42, but not non-traditional ideology and childcare at age 30, was associated with decreased risk of mental ill-health symptoms at age 42. A contributory explanation could be that detrimental effects of gender inequality on mental health emerge from accumulation over time [46]. Ultimately, however, the later adulthood findings support our assumption of net benefits of gender equality for mental health; for men by, for example, adding healthier relationships to partner and children [15,16], and for women by, for example, loosened multiple demands [48], and for both genders from sharing life more equally as a whole [20]. In other words, our results give no support for the idea that departing from traditional positions of masculinity (for men) and femininity (for women) is detrimental to mental health [28,29].

The results stratified by gender show that women, but not men, who report a non-traditional gender ideology have a 63% decreased risk of anxious symptoms. Could it be that resistance to societal change, including gender equality progress, is related to nervousness and anxiety in general? Furthermore, acknowledging the asymmetric dimensions of the gender system [10,49], women who reject a gender-equal society disagree with an improved societal position for themselves, whilst men who reject a gender-equal society may want to defend their societal dominance [28]. In fact, this more reasonable men’s position may partly explain why we found no association between a traditional gender ideology and mental ill-health symptoms among men.

The general pattern in the bivariate analyses does not support our assumption of a converged gender gap in mental ill-health from increased gender equality, in terms of women benefiting more than men. It would be fairer to propose that the genders are differently affected; women benefit in anxious symptoms from experiencing a non-traditional partnership at age 42, while men benefit in anxious symptoms from a non-traditional childcare division at age 42. These findings are supported by earlier research [13,14]. For men, practising an equal or shifting tradition in childcare was particularly beneficial when not having a partner, which validates the policy relevance of gender equality efforts also when a parental couple is separated.

### Analytical findings

For women, the combinational analyses adjusted for health and social confounding showed that reporting a non-traditional ideology at age 30, combined with traditional as well as non-traditional childhood, was associated with decreased risks of anxious symptoms (76% and 78%, respectively). For men, reporting a non-traditional childcare at age 42, combined with traditional as well as non-traditional childhood, was associated with decreased risks of depressive symptoms (84% and 78%, respectively) (all estimates compared to the reference combination of traditional adulthood/traditional childhood).

This confirms our assumption that gender equality benefits mental health among both women and men. Our results among the women may be theoretically understood in relation to socioeconomic advancement and loosened multiple demands, while our results among the men may be related to the theories of expanded life roles, and childcare as beneficial per se for mental health. They also support our proposal above that women and men are affected by different aspects of gender (in)equality, but not our expectation of an interaction between inherited and one’s own gendered life on mental ill-health [14,39]. A further comment is that the only statistically significant results for women are related to anxious symptoms, whereas for men it is depressive symptoms. These findings may be regarded as at odds with our earlier research, in which we drew conclusions about a reversed gender bias in the expression of depressive symptoms in the Western world [42]. The diagnostic criteria of depressive symptoms have been derived from research almost exclusively on women. Thus, men’s whole range of expression of depressive symptoms may not be captured in traditional questionnaires.

The threefold increased risk of anxious symptoms among women who reported traditional gendered childhood in combination with non-traditional childcare at age 30 is not statistically significant (3.09, CI: 0.90–10.62), but interesting. It indicates that gender equality, in terms of departing from firm femininity ideals around early motherhood, could be detrimental for women’s mental health [40]. It also indicates that continuing or opposing one’s gendered history may have a unique impact on mental health [41]. In other words, self-reported anxiety from acting against the gendered tradition of childcare duty is offset by having had a mother with working life ambition/necessity. In the terminology of role theory, failing to live up to expectations from one’s family of origin may produce role strain due to incomplete guidance from the mother and feeling alone in constructing one’s own motherhood [35-37]. It is notable that being non-traditional in childcare at age 42 includes no association with anxious symptoms; a simple interpretation is that the femininity ideals of caring are stronger in early motherhood (the child on average 5 years old) than in later motherhood (the child on average 17 years old).

### Strengths and weaknesses

The main strengths of the present study are that the cohort study gives an opportunity to analyse a life-course perspective on gendered life during a 26-year period with an extremely high response rate. Acknowledging the development of gender equality during recent decades, it is also an advantage that the studied individuals were born in the same year (1965).

Even though the combinational analyses were adjusted for baseline mental health position, and for socioeconomic/family situation, one cannot exclude the risk of residual confounding in the associations between gendered life and mental ill-health. Another weakness is the lack of statistical power when categorising individuals into traditional or non-traditional, and when stratifying the analyses by gender. This includes that the tests of interaction between gender and gendered life on mental ill-health could generally not be statistically verified.

The measures of anxious and depressive symptoms also need to be discussed. All measures of mental health problems are self-rated. Even clinical diagnoses of psychiatric disorder are based on subjective symptoms rather than objective measures. Our intention was not to measure psychiatric diagnosis, but to capture self-perceived anxiety and depression via direct questions about the frequency of a set of indicative symptoms. From Sweden it has been reported that mental ill-health, indicated by self-reported anxiety and register data on psychiatric care for anxiety, displays coherence by gender and over time [6]. Nevertheless, we advise against transferring our findings regarding the influence of gendered life on mental ill-health symptoms to more severe and clinically diagnosed mental health problems. Further, even though experiences regarding the mother’s paid work position during childhood may be wrong (e.g. reporting a homemaking and childrearing mother, because she was at home before and after school, although working part-time in between), individuals’ memories of their gendered childhood are the key aspect in the present study.

Finally, women and men report large differences in gendered life, particularly regarding the division of childcare duties. Since the population does not include parental couples, it is not possible to test whether the high proportion of women doing most/all corresponds to a similarly high proportion among their parental partners doing less/nothing (in contrast to the low proportion among men in the Northern Swedish Cohort). However, this is not probable; i.e. it seems reasonable to assume that women’s and men’s perceptions of gendered life are in fact inconsistent [30,34]. On objective grounds, this represents a fault (the reports on nothing, less, most, and all could in principle be empirically tested); on subjective grounds, this is correct (as long as the gender system exists, women and men will have different views and experiences of it) [7]. Our study considered subjective aspects of gendered life. It must be considered that objective measures, such as observational time studies and parental leave uptake, may show other associations with mental ill-health symptoms.

## Conclusion

This study reports that parents’ gendered life is not associated with a person’s own gendered life; in fact, there was a tendency that a homemaking and childrearing mother was associated with claiming an ideology of gender-equal society. Furthermore, opposing or continuing one’s gendered history generally did not affect the associations between adulthood gender position and self-reported mental ill-health. The main findings were that women benefit in terms of reduced anxious symptoms from a more gender-equal ideology at age 30, whilst men benefit in terms of reduced depressive symptoms from a more gender-equal childcare at age 42. An opposing indication, in two ways, is that women who were equal or non-traditional in childcare at age 30 had an increased risk of anxious symptoms, but only if they experienced a gender-traditional childhood. Future studies on the potential interaction between mental health and gendered life in the family of origin and the family of maturity, stratified by gender, are welcomed.

## Competing interests

The authors declare that they have no competing interests.

## Authors’ contribution

AM performed the statistical analyses and wrote the first draft of the manuscript. MN was responsible for the theoretical and empirical aspects of the exposures of gender. AH is PI of the Northern Swedish cohort, and as such responsible for the collection and maintenance of all data. All authors contributed to the intellectual work, choice of methods, interpretation of results, and style of writing. All authors finally read and approved the present version of the manuscript.

## Pre-publication history

The pre-publication history for this paper can be accessed here:

http://www.biomedcentral.com/1471-2458/12/493/prepub
